# Robust anti‐nociceptive effects of monoacylglycerol lipase inhibition in a model of osteoarthritis pain

**DOI:** 10.1111/bph.13574

**Published:** 2016-09-23

**Authors:** James J Burston, Paul I Mapp, Sarir Sarmad, David A Barrett, Micah J Niphakis, Benjamin F Cravatt, David A Walsh, Victoria Chapman

**Affiliations:** ^1^ Arthritis Research UK Pain CentreUniversity of Nottingham, Medical School, Queen's Medical CentreNottinghamUK; ^2^ School of Life SciencesUniversity of Nottingham, Medical School, Queen's Medical CentreNottinghamUK; ^3^ Centre for Analytical Bioscience, School of PharmacyUniversity of NottinghamNottinghamUK; ^4^ The Skaggs Institute for Chemical Biology and Department of Chemical PhysiologyThe Scripps Research InstituteLa JollaCAUSA; ^5^ Committee of Neurobiology of Addictive DisordersThe Scripps Research InstituteLa JollaCAUSA

## Abstract

**Background and Purpose:**

Chronic pain is often a symptom of knee osteoarthritis (OA) for which current analgesics are either inadequate or are associated with serious side effects. The endocannabinoid system may offer alternative targets for pain relief. We evaluated the effects of a potent and selective monoacylglycerol (MAG) lipase inhibitor (MJN110) on OA pain behaviour, spinal mechanisms of action and joint histopathology in the rat.

**Experimental Approach:**

Intra‐articular injection of monosodium iodoacetate (MIA) models OA pain and mimics clinical joint pathology. Effects of MJN110 on MIA‐induced weight‐bearing asymmetry and lowered paw withdrawal thresholds (PWTs), changes in spinal gene expression and brain levels of relevant lipids were determined.

**Key Results:**

Acute MJN110 (5 mg·kg^−1^) significantly reversed MIA‐induced weight‐bearing asymmetry (MIA/vehicle: 68 ± 6 g; MIA/MJN110: 35 ± 4 g) and lowered ipsilateral PWTs (MIA/vehicle: 7 ± 0.8 g; MIA/MJN110: 11 ± 0.6 g), via both CB_1_ and CB_2_ receptors. Repeated treatment with MJN110 (5 mg·kg^−1^) resulted in anti‐nociceptive tolerance. A lower dose of MJN110 (1 mg·kg^−1^) acutely inhibited pain behaviour, which was maintained for 1 week of repeated administration but had no effect on joint histology. MJN110 significantly inhibited expression of membrane‐associated PGE synthase‐1 in the ipsilateral dorsal horn of the spinal cord of MIA rats, compared with vehicle‐treated MIA rats. Both doses of MJN110 significantly elevated brain levels of the endocannabinoid 2‐arachidonoylglycerol.

**Conclusions and Implications:**

Our data support further assessment of the therapeutic potential of MAG lipase inhibitors for the treatment of OA pain.

**Abbreviations**
2‐AG: 2‐arachidonoylglycerol
AA: arachidonic acid
GFAP: glial fibrillary acidic protein
MAG lipase: monoacylglycerol lipase
MIA: monosodium iodoacetate
mPGES1: membrane‐associated PGE synthase‐1
OA: osteoarthritis

## Tables of Links

**Table:** 

**TARGETS**	
**GPCRs** ^*a*^	**Enzymes** ^*b*^
CB_1_ receptors	mPGES1, membrane‐bound PGE synthase‐1
CB_2_ receptors	MGL, monacylglycerol lipase
	COX‐2

**LIGANDS**
2‐AG, 2‐arachidonoylglycerol
Rimonabant, SR141716A
SR144528

These Tables list key protein targets and ligands in this article that are hyperlinked to corresponding entries in http://www.guidetopharmacology.org, the common portal for data from the IUPHAR/BPS Guide to PHARMACOLOGY (Southan *et al.*, 2016), and are permanently archived in the Concise Guide to PHARMACOLOGY 2015/16 (^*a,b*^Alexander *et al.*, 2015a,b).

## Introduction

Osteoarthritis (OA) is one of the most common joint diseases (Zhang *et al.,* 2008) and is associated with loss of articular cartilage, synovitis and changes in peri‐articular and subchondral bone (Goldring and Goldring, 2007). Pain is the major symptom of OA (Chan *et al.,* 2014), and its relief by current analgesics is often limited by short duration of action, or unwanted side effects. For example, despite the promise shown by COX‐2 inhibitors to reduce both inflammation and pain in human OA, adverse cardiovascular effects (Juni *et al.,* 2004) have limited their therapeutic benefit. Total knee joint replacement remains the only successful treatment option for many OA sufferers, indicating a pressing need for new analgesics (Glyn‐Jones *et al.,* 2015).

The endogenous cannabinoid system (ECS), which is composed of two well‐characterized receptors (CB_1_ and CB_2_), endogenous ligands and several biosynthetic and metabolic enzymes, is increasingly recognized for its ability to modulate pain and inflammation, as well as its pharmacological tractability. The ECS modulates nociceptive signalling in numerous preclinical models of pain (see Rani Sagar *et al.,* 2012). Clinical applications of direct CB_1_ receptor agonists indicate that ECS‐targeting agents do offer therapeutic benefit for the treatment of pain in preclinical pain models, as well as in different human pain states (Elikkottil *et al.,* 2009). The ECS also has robust anti‐inflammatory actions and anxiolytic effects, which may be beneficial in reducing emotional and affective components of chronic pain (Smith and Zautra, 2008; Sinikallio *et al.,* 2014).

Levels of endocannabinoids are elevated by noxious stimuli such as OA pain, in key areas involved in pain processing, including the spinal cord (Woodhams *et al.,* 2015). One promising approach for harnessing the analgesic potential of the ECS is to target the catabolic enzymes that regulate levels of endocannabinoids. To date, there has been a predominant focus on protecting levels of the endocannabinoid anandamide (AEA) by the inhibition of fatty acid amide hydrolase (FAAH); however, clinical benefit has yet to be demonstrated (see Rani Sagar *et al.,* 2012) and a clinical trial of an FAAH inhibitor for OA pain reported no positive benefit over placebo (Huggins *et al.,* 2012). Unlike AEA, the endocannabinoid 2‐arachidonoylglycerol (2‐AG) is a full efficacy agonist at CB_1_ and CB_2_ receptors (Savinainen *et al.,* 2001) and activates both CB_1_ and CB_2_ receptors with similar potency. Exogenous 2‐AG has robust anti‐nociceptive effects in models of acute and chronic pain (Woodhams *et al.,* 2015). Monoacylglycerol lipase (MAG lipase) plays a pivotal role in the degradation of 2‐AG, and inhibition of this enzyme results in a significant elevation in 2‐AG in peripheral and central nervous tissue (Long *et al.,* 2009). MAG lipase activity also appears to be intricately involved in the generation of pro‐inflammatory PGs in the CNS (Nomura *et al.,* 2011). MAG lipase inhibitors have anti‐nociceptive effects in models of both acute and chronic pain states (see Ignatowska‐Jankowska *et al.,* 2015). The potential effects of MAG lipase inhibitors on OA pain, and whether effects are maintained upon sustained treatment, are yet to be described.

Intra‐articular injection of the metabolic inhibitor monosodium iodoacetate (MIA) into the knee joint leads to disruption of chondrocyte glycolysis, loss of articular cartilage and subchondral bone remodelling, similar to the pathology associated with human OA (Sagar *et al.,* 2010). Functionally, this model of OA joint damage leads to decreased weight‐bearing on the injured knee, as seen in human OA (Christiansen and Stevens‐Lapsley, 2010), as well as characteristics of central sensitization, including distal allodynia, activation of spinal microglia and astrocytes and hyper‐excitability of spinal neurones, (Sagar *et al.,* 2013). Previously, we have reported increased expression of spinal CB_2_ receptors and increased function in the MIA model of OA pain (Burston *et al.,* 2013). The aims of the present study were to quantify the effects of acute and of repeated administration of a selective MAG lipase inhibitor 2,5‐dioxopyrrolidin‐1‐yl 4‐(bis(4‐chlorophenyl)methyl)piperazine‐1‐carboxylate (MJN110) on pain behaviour and joint pathology and to evaluate potential mechanisms of effect at the level of the spinal cord.

## Methods

### Animals

All animal care and experimental procedures were in accordance with UK Home Office Animals (Scientific Procedures) Act (1986) and the International Association for the Study of Pain. Animal studies are reported in compliance with the ARRIVE guidelines (Kilkenny *et al.*, 2010; McGrath and Lilley, 2015). Experiments were conducted in a blinded fashion (coding was carried out by another member of the research group), such that the person conducting pain behaviour assessment was unaware of injury status or drug treatment of the rats. Adult male rats (70 Sprague Dawley rats, weight 180–200 g; Charles River, UK) were used in these studies.

### Intra‐articular injections and behavioural testing

Rats were anesthetized (isoflurane 2.5–3% in 100% O_2_), and once areflexic received a single intra‐articular injection of 1 mg MIA (Sigma, Gillingham, Dorset, UK) in 50 μL saline or 50 μL saline (control rats) through the infra‐patellar ligament of the left knee (Sagar *et al.,* 2010). Pain behaviour was assessed at baseline (prior to injection, day 0). Weight distribution on the left (ipsilateral) and right (contralateral) hindlimb was assessed using an incapacitance tester (Linton Instrumentation Diss, Norfolk, UK), as previously described (Sagar *et al.,* 2010). Changes in hindpaw withdrawal thresholds were assessed using von Frey monofilaments, Linton Instrumentation Diss, Norfolk, UK (Semmes‐Weinstein monofilaments of bending forces 0.4–26 g) as previously described (Sagar *et al.,* 2010). The lowest weight filament to elicit a withdrawal reflex was taken as the hindpaw withdrawal threshold.

### Acute administration of MJN110

Pain behaviour was quantified on days 3, 7, 14 and 21 post‐MIA injection. At day 21, MIA‐injected rats were stratified (by an independent experimenter) into two groups based on pain behaviour over the previous weeks. On day 28, baseline pain behaviour was quantified, and then rats received an i.p. injection of either vehicle (5% ethanol, 5% emulphor and 90% sterile saline) or MJN110 (5 mg·kg^−1^) in vehicle. Three hours later, pain behaviour was quantified as described previously.

### The contribution of CB_1_ and CB_2_ receptors to the anti‐nociceptive effects of MJN110

Pain behaviour was quantified on days 3, 7, 10, 14 and 17 post‐MIA injection. At day 17, MIA‐injected rats were stratified (by an independent experimenter) into three groups based on previous pain behaviour. On day 21, baseline pain behaviour was quantified, and then rats received an i.p. injection of either vehicle (5% ethanol, 5% emulphor and 90% sterile saline), or the CB_1_ receptor antagonist rimonabant (SR141716A; 3 mg·kg^−1^), or the CB_2_ receptor antagonist SR144528 (3 mg·kg^−1^), followed 30 min later by a second i.p. injection of MJN110 (5 mg·kg^−1^). Three hours later, pain behaviour was quantified as described earlier. Doses of antagonists were based on previous work (Ignatowska‐Jankowska *et al.,* 2015).

### Repeated administration of MJN110

Pain behaviour was quantified on days 3, 7, 10, 14 and 17 post‐MIA injection. On day 17, rats were stratified based on pain behaviour. Baseline pain behaviour was quantified on day 21 and rats received either an i.p. injection of vehicle or MJN110 (1 or 5 mg·kg^−1^). Three hours later, pain behaviour was quantified. Doses of MJN110 were based on activity‐based protein profiling that demonstrated maximal reduction in activity‐based probe binding to MAG lipase by 5 mg·kg^−1^ of MJN110, suggesting complete inhibition of MAG lipase activity, whereas the 1 mg·kg^−1^ of MJN110 produced a sub‐maximal reduction of MAG lipase probe binding. Rats then received daily i.p. injections of vehicle or MJN110 (1 or 5 mg·kg^−1^) until day 28, and pain behaviour was quantified on days 24 and 28.

### Tissue collection

Four hours following the final dose of MJN110, rats were killed by cranial concussion followed by exsanguination. Spinal cord and brain were dissected and snap frozen by immersion in liquid nitrogen. Knee joints were collected and stored in neutral buffered formalin.

### RNA extraction and cDNA synthesis

Samples of frozen ipsilateral dorsal spinal cord tissue (about 25 mg each) were homogenized in 2 mL of ice cold Tri reagent (Sigma‐Aldrich, Gillingham, Dorset, UK) and RNA isolated via separation of aqueous phase from DNA and protein phases, followed by precipitation in a sodium acetate and isopropanol solution before being dissolved in RNAS free water, as previously described (Okine *et al.,* 2012). For cDNA synthesis, RNA was reverse transcribed as previously described (Okine *et al.,* 2012), using Superscript III reverse transcriptase (Life Technologies, Paisley, UK).

### Taqman quantitative real‐time PCR

Gene expression was quantified utilizing the relative standard curve method, based on Taqman quantitative real‐time PCR, as previously described (Okine *et al.,* 2012). Primer and probe sequences were either taken from published work (Okine *et al.,* 2012) or designed (see Table 1 for sequences) using primer express version 3 (Life Technologies, UK) and synthesized by MWG Biotech, (Ebersberg, Germany). Samples were run in triplicate, and coefficient of variation for all triplicates was less than 10%; triplicate values were then averaged to produce a single value per rat. Data are expressed as a ratio of gene expression levels with reference to β‐actin.

**Table 1:** Sequences for real‐time PCR primers and probes

Gene	Forward primer	Reverse primer	TAQMAN probe
GFAP	5‐TGGCCACCAGTAACATGCAA‐3	5‐CAGTTGGCGGCGATAGTCAT‐3	5‐CAGACGTTGCTTCCCGCAACGC‐3
mPGES1	5‐GCGAACTGGGCCAGAACA‐3	5‐GGCCTACCTGGGCAAAATG‐3	5‐CCCCGGAGCGAATGCGTGG3
COX‐1	5‐GGCTGGCCGGATTGGT‐3	5‐ATTTCTCGGGACTCCTTGATGA‐3	5‐AACTTTGACTACCATGTTCTGCATGTGGCC‐3

### Knee histology

Tibiofemoral joints were removed and postfixed in neutral buffered formalin (Sagar *et al.,* 2010), decalcified in EDTA and sectioned for staining with haematoxylin and eosin. The pathology scoring was performed on stained sections, as previously described (Sagar *et al.,* 2014).

Chondropathy was scored (Janusz *et al.,* 2002)on a scale of 0–5 as follows: 0, cartilage of normal appearance; 1, minimal fibrillation, superficial zone only; 2, mild, extends to the upper middle zone; 3, moderate, well into the middle zone; 4, marked, into the deep zone but not to the tidemark; and 5, severe, full thickness degeneration to tidemark. Cartilage damage was estimated as the proportion of the section of the medial tibial plateaux involved, 1/3, 2/3 or 3/3, and the cartilage score multiplied by 1, 2 or 3 respectively to give a total chondropathy score.

Synovial inflammation was scored according to the thickness of the synovial lining layer and synovial cellularity in the medial and lateral tibiofemoral compartment (Mapp *et al.,* 2008) and was graded on a scale from 0 (lining cell layer 1–2 cells thick) to 3 (lining cell layer >9 cells thick and/or severe increase in cellularity).

### Measurement of 2‐AG, PGE_2_ and arachidonic acid (AA) in brain samples

Targeted LC‐MS/MS was used for analysis of PGE_2_, AA and 2‐arachidonoyl glycerol (2‐AG) in rat brain. Samples, previously stored at −80°C, were thawed on ice and weighed accurately; internal standards 2‐AG‐d8 (10 μM), AA‐d8 (10 μM) and PGD_2_‐d4 (1 μM) and butylated hydroxytoluene (100 μL, 0.5%) were added before extraction with ethyl acetate : hexane (9:1 *v/v*). Tissue homogenates were vortex‐mixed, centrifuged (5000× *g* for 15 min at 4°C), and the resulting supernatants were evaporated to dryness. Tissue extracts were reconstituted in 200 μL of acetonitrile : water (1:1), and 10 μL was injected for analysis by LC‐MS/MS using an Agilent 1100 series (Agilent Technologies, Waldbronn, Germany) coupled to a Micromass Quattro Ultima^tm^ triple quadrupole mass spectrometer (Waters, Manchester, UK) equipped with electrospray negative ionization. The column used was an ACE Excel 3 μm particle diameter, Super C18, 150 × 2.1 mm at 40°C, and the mobile phase was a linear gradient of water +0.02% formic acid (mobile phase A) and acetonitrile : methanol (4:1) +0.02% formic acid (mobile phase B). Flow rate was 300 μL·min^−1^, and analysis time was 15 min. Quantification of the PGE_2_, AA and 2‐AG was done using fully extracted calibration standards for each of the analytes and was performed using QuanLynx v 4.1, Waters, Manchester, UK.

### Data and statistical analysis

The data and statistical analysis in this study comply with the recommendations on experimental design and analysis in pharmacology (Curtis *et al*., 2015). Results are shown as means ± SEM. All statistics were calculated using Prism 5.0 software (Graphpad, La Jolla, USA). Data were analysed with either one‐way or two‐way anova, *t*‐test, or Spearman's correlation. Data were considered significant if *P* values were less than 0.05. For data that did not pass normality testing, non‐parametric statistics were used (Kruskal–Wallis one‐way ANOVA). Correlations between rat spinal gene expression and pain behaviour were determined with a Pearson correlation. To evaluate whether the anti‐nociceptive efficacy of MJN110 was reduced over time, regression analysis determined whether there was a significant difference (*P* < 0.05) from zero in the slope of the pain behaviour in the presence of repeated MAG lipase inhibition; a significant difference indicated a reduction in inhibition of pain behaviour.

### Materials

MJN110 was a kind gift from Micah Niphakis and Benjamin Cravatt. MIA, Emulphore and Ethanol was purchased from Sigma (Gillingham, Dorset, UK) were purchased from Sigma, UK. Rimonabant and SR144528 were from Cayman Chemical Ann Arbor, Michigan, USA.

## Results

### Acute MAG lipase inhibition reverses established MIA‐induced pain behaviour

**Figure 1:**
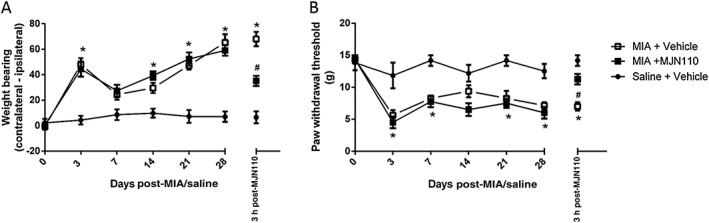
Acute administration of MJN110 reverses established MIA‐induced pain behaviour. Intra‐articular injection of MIA was associated with a significant increase in weight‐bearing asymmetry (A) and lowered hindpaw withdrawal thresholds (B). Acute systemic administration of the MAG lipase inhibitor MJN110 (5 mg·kg^−1^; administered on day 28) attenuated MIA‐induced changes in weight‐bearing asymmetry (A) and lowered hindpaw withdrawal thresholds (B), as denoted by the data point listed as 3 h post‐MJN110. Data shown are means ± SEM; *n* = 6 saline groups; *n* = 8 rats MIA groups; total rats used = 22. *P < 0.05, significant difference between MIA + vehicle and saline + vehicle; #*P* < 0.05, significant difference between MIA + vehicle versus MIA + MJN110; two‐way ANOVA and Bonferroni *post hoc* test.

As previously described (Sagar *et al.,* 2010), intra‐articular injection of MIA was associated with significant decreases in weight‐bearing on the ipsilateral hindlimb and lowered mechanical withdrawal thresholds of the ipsilateral hindpaw, compared with saline‐treated rats (Figure 1A, B). Acute treatment with 5 mg·kg^−1^ of MJN110 significantly reversed established MIA‐induced weight‐bearing asymmetry and increased hindpaw withdrawal thresholds 3 h post‐administration (Figure 1A, B).

### Contributions of CB_1_ and CB_2_ receptors to MJN110‐mediated inhibition

**Figure 2:**
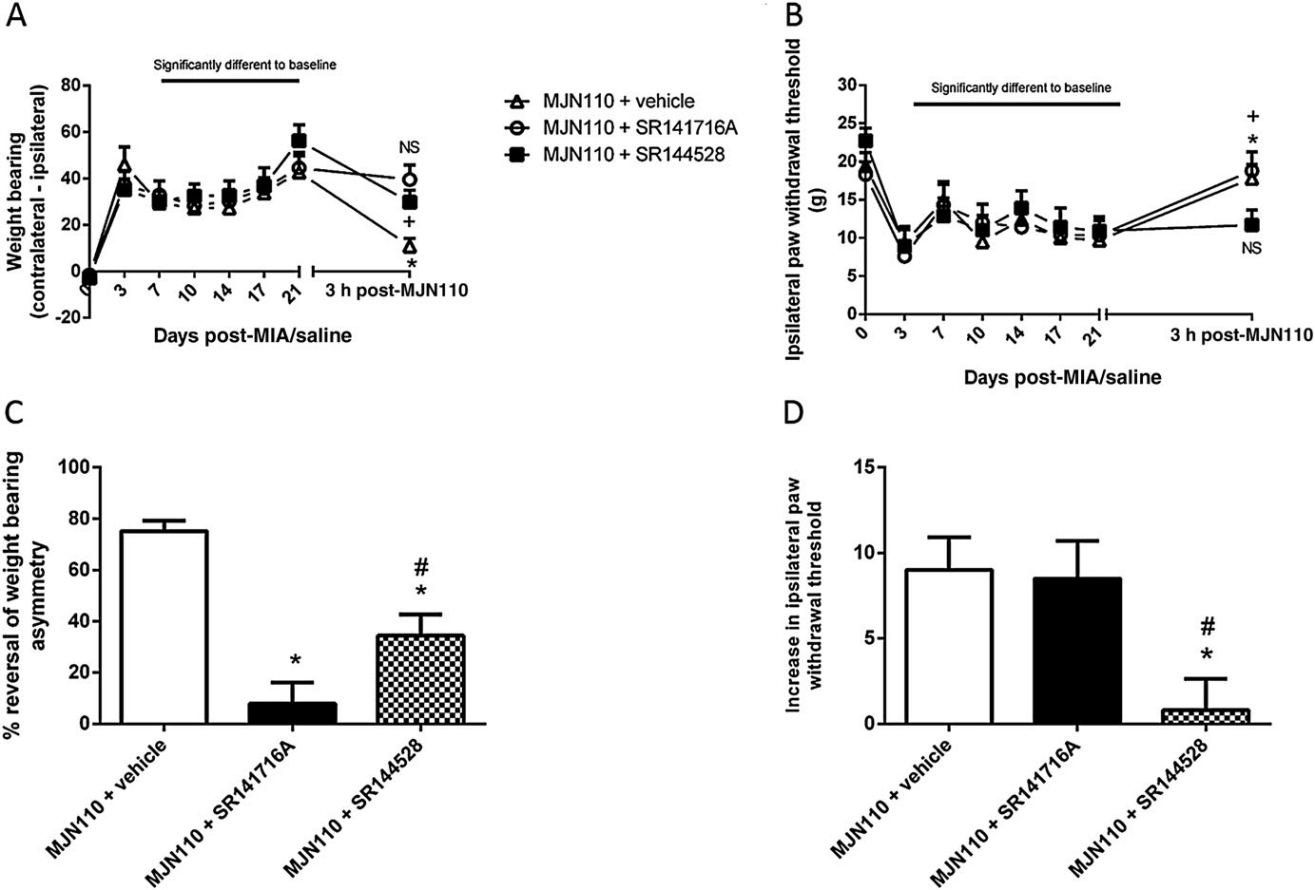
Effect of CB receptor blockade on MJN110‐mediated analgesia. (A) Intra‐articular injection of MIA was associated with a significant increase in weight‐bearing asymmetry, which was reversed by a single administration of 5 mg·kg^−1^ MJN110; this effect was blocked by the CB_1_ receptor antagonist SR141716A (3 mg·kg^−1^) and the CB_2_ receptor antagonist SR144528 (3 mg·kg^−1^). Data are mean + SEM; *n* = 10 rats per group. *P < 0.05, significant difference between MJN110 + vehicle group pre‐ and post‐drug on day 21; ^+^ *P* < 0.05, significant difference between MJN110 + SR144528 pre‐ and post‐drug on day 21; NS = no significant difference between MJN110 + SR141716A pre‐ and post‐drug on day 21; two‐way ANOVA and a Bonferroni *post hoc* test. (B) Intra‐articular injection of MIA was associated with a significant decrease in ipsilateral paw withdrawal thresholds, which was reversed by a single administration of 5 mg·kg^−1^ MJN110; this effect was blocked by the CB_2_ receptor antagonist SR144528 (3 mg·kg^−1^) but not the CB_1_ receptor antagonist SR141716A (3 mg·kg^−1^). Data shown are means ± SEM; *n* = 10 rats per group. *P < 0.05, significant difference between MJN110 + vehicle group pre‐ and post‐drug on day 21; ^+^ *P* < 0.05, significant difference between MJN110 + SR141716A group pre‐ and post‐drug on day 21; NS = no significant difference between MJN110 + SR144528 group pre‐ and post‐drug on day 21; two‐way ANOVA and Dunns *post hoc* test. (C) Analysis conducted on transformed weight‐bearing data confirmed that the effects of MJN110 on MIA‐induced weight‐bearing asymmetry were blocked by SR141716A and SR144528. The blockade by SR144528 was less than that produced by SR141716A. Data shown are means ± SEM; *n* = 10 rats per group. *P < 0.05, significantly different from MJN110 + Vehicle group, ^#^ *P* < 0.05, significantly different from MJN110 + SR141716A group; two‐way ANOVA and Bonferroni *post hoc* test. (D) Analysis conducted on transformed ipsilateral hindpaw withdrawal threshold data (increase in paw withdrawal threshold relative to pre‐drug at day 21) confirmed that the effects of MJN110 were blocked by SR144528, but not SR141716A. Data shown are means ± SEM; *n* = 10 rats per group. *P < 0.05, significantly different from MJN110 + Vehicle group, ^#^ *P* < 0.05, significantly different from MJN110 + SR141716A group; one‐way anova followed by Dunn's *post hoc* test.

The ability of CB_1_ or CB_2_ receptor antagonists to block the anti‐nociceptive effects of MJN110 on pain behaviour was investigated. The anti‐nociceptive effects of MJN110 (5 mg·kg^−1^) on weight‐bearing asymmetry were blocked by the CB_1_ receptor antagonist SR141716A (Figure 2A, C). The CB_2_ receptor antagonist SR144528 also reduced the anti‐nociceptive effects of MJN110 on weight‐bearing asymmetry (Figure 2A, C), but the magnitude of the reversal was significantly less than that achieved by SR141716A. MJN110‐mediated reversal of MIA‐induced lowering of ipsilateral hindpaw withdrawal thresholds was significantly blocked by the CB_2_ receptor antagonist SR144528, but not the CB_1_ receptor antagonist (Figure 2B, D).

### Anti‐nociceptive tolerance following repeated administration of high‐dose MJN110

**Figure 3:**
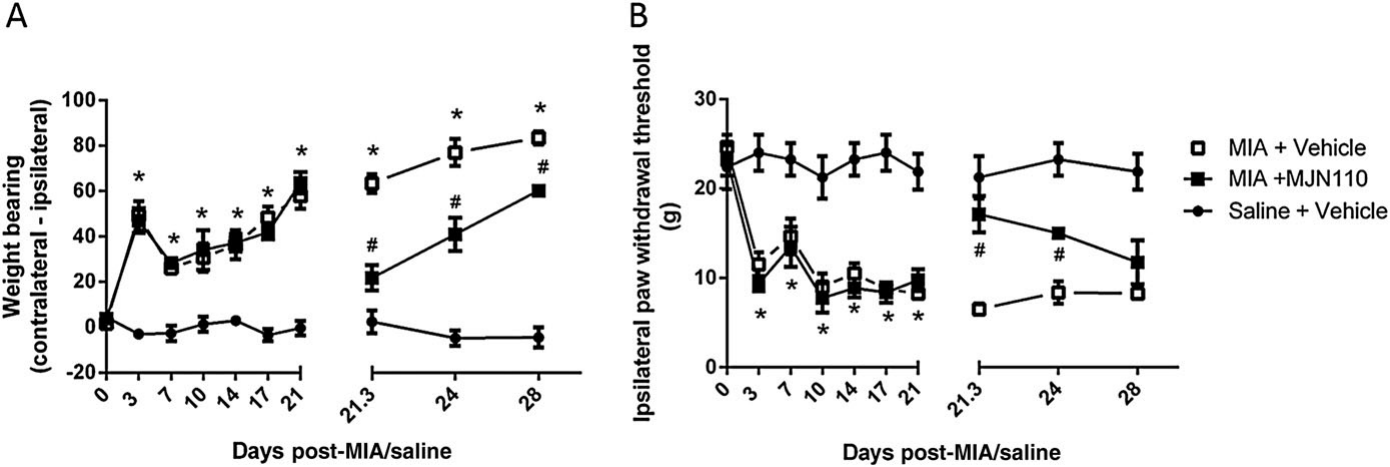
Loss of the anti‐nociceptive effects of 5 mg·kg^−1^ MJN110 following repeated administration. Intra‐articular injection of MIA was associated with a significant increase in weight‐bearing asymmetry (A) and lowered hindpaw withdrawal thresholds (B). Repeated administration of the MAG lipase inhibitor MJN110 (5 mg·kg^−1^; administered on days 21–28) attenuated MIA‐induced changes in weight bearing and lowered hindpaw withdrawal thresholds; however, the magnitude of inhibition declined over the week of treatment. Data shown are means ± SEM; *n* = 8 (total rats used =24). *P < 0.05, significant difference between MIA + vehicle and saline + vehicle groups; ^#^ *P* < 0.05, significant difference between MIA + vehicle and MIA + MJN110; two‐way ANOVA and Bonferroni *post hoc* test.

Although the first injection of 5 mg·kg^−1^ MJN110 had robust anti‐nociceptive effects on pain behaviour (Figure 3A, B), daily i.p. injections from days 21 to 28 post‐MIA injection induced tolerance to these anti‐nociceptive effects. Regression analysis of the three time points of MJN110 dosing (days 21, 24 and 28) showed that the anti‐nociceptive effects of MJN110 on MIA‐induced weight bearing were significantly reduced over time (*F* = 6345). This was also true for the effects of repeated MJN110 treatment on hindpaw withdrawal thresholds (*F* = 15 392, *P* < 0.05).

### Repeated low‐dose MJN110 exhibits sustained anti‐nociceptive effects on pain behaviour and reduces spinal expression of mPGES1

**Figure 4:**
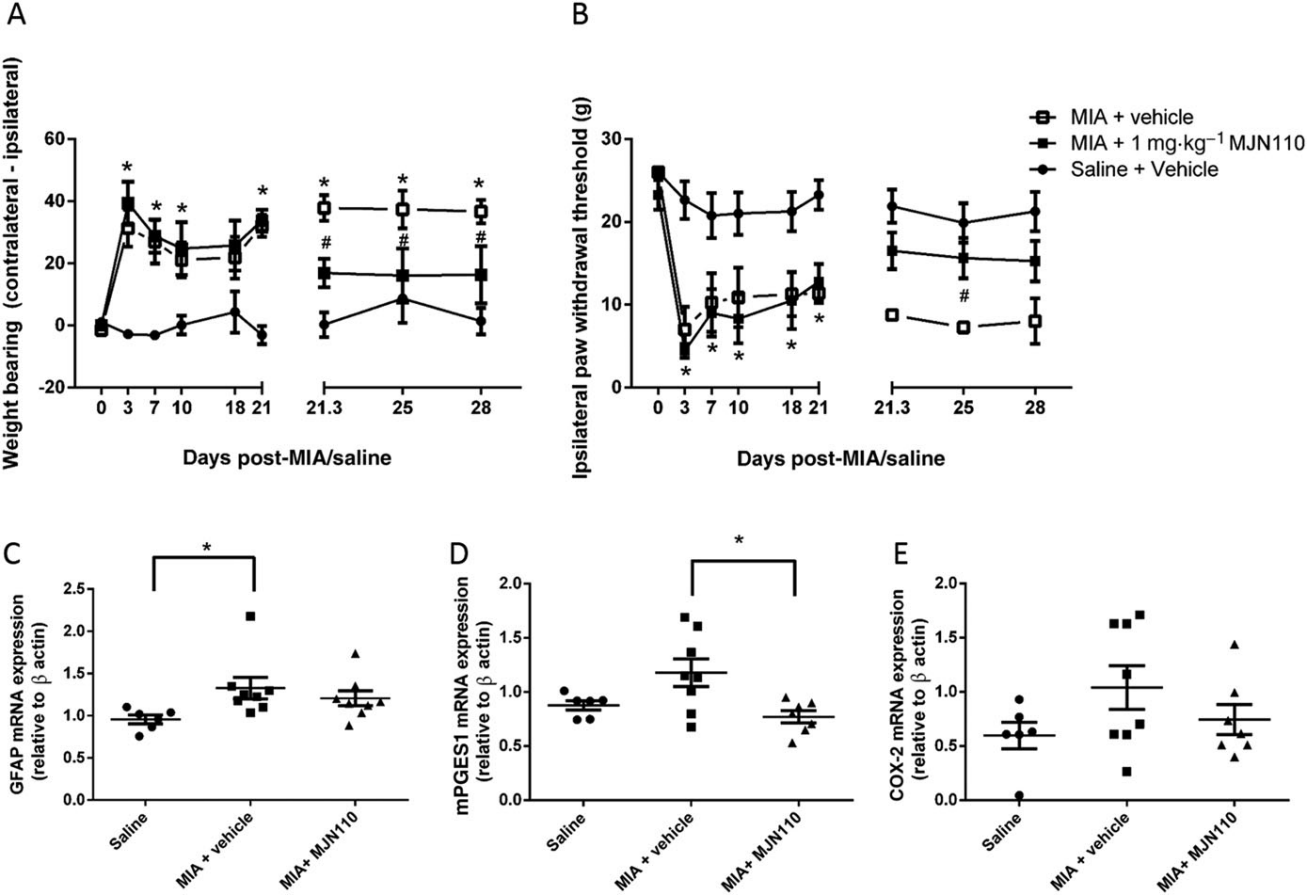
Repeated administration of 1 mg·kg^−1^ MJN110 had sustained anti‐nociceptive effects on pain behaviour and altered markers of spinal sensitization. Intra‐articular injection of MIA was associated with a significant increase in weight‐bearing asymmetry (A) and lowered hindpaw withdrawal thresholds (B). Repeated injection of MJN110 (1 mg·kg^−1^; administered daily from days 21 to 28) attenuated MIA‐induced changes in weight‐bearing and lowered hindpaw withdrawal thresholds. Data shown are means ± SEM; *n* = 8 rats per group (total rats used = 24). *P < 0.05, significant difference between MIA + vehicle and saline + vehicle groups; ^#^ *P* < 0.05, significant difference between MIA + vehicle and MIA + MJN110; two‐way ANOVA and Bonferroni *post hoc* test. (C) Intra‐articular injection of MIA was associated with a significant increase in spinal GFAP mRNA, compared with saline‐injected rats. Repeated administration of MJN110 (1 mg·kg^−1^) did not significantly alter MIA‐induced increases in spinal GFAP expression in the spinal cord. (D) There was no difference in spinal mPGES1 mRNA expression in MIA versus saline rats. Repeated administration of MJN110 (1 mg·kg^−1^) significantly lowered spinal mPGES1 mRNA expression in MIA‐treated rats (D) compared with the MIA vehicle group**.** (E ) Spinal COX‐2 mRNA expression was comparable between the three groups. *P < 0.05, significantly different as indicated; two‐way ANOVA and Bonferroni *post hoc* test.

To determine if prolonged exposure to sub‐maximal doses of MJN110 produced a sustained inhibition of pain behaviour; the effects of repeated dosing with 1 mg·kg^−1^ of MJN110 were investigated. This dose of MJN110, which was previously shown to partly inhibit brain MAG lipase in rats (Niphakis *et al.,* 2013), produced a significant inhibition of pain behaviour (Figure 4A, B). Regression analysis of the three time points of MJN110 dosing showed the anti‐nociceptive effects of this lower dose of MJN110 on MIA‐induced weight bearing (*F* = 0.003719, DFn = 1.00, DFd = 22.00), and hindpaw withdrawal thresholds (*F* = 0.1477, DFn = 1.00, DFd = 22.00) did not vary over time. As this dose of MJN110 had sustained anti‐nociceptive effects on pain behaviour until day 28, potential effects of the intervention on the spinal expression of pro‐inflammatory markers and joint pathology were evaluated.

Intra‐articular injection of MIA induced a significant increase in the expression of the astrocyte marker glial fibrillary acidic protein (GFAP) mRNA. MJN110 treatment did not significantly alter MIA‐induced up‐regulation of GFAP mRNA. There was, however, a significant difference in the expression of membrane‐associated PGE synthase‐1 (mPGES1) in the MIA MJN110 group and the MIA vehicle group (Figure 4D). Correlation analysis revealed that spinal GFAP expression was correlated with weight‐bearing asymmetry (R^2^ = 0.2055) and inversely correlated with paw withdrawal threshold (R^2^ = 0.2061). Spinal mPGES1 expression was correlated with weight‐bearing asymmetry (R^2^ = 0.2055). Spinal COX‐1 expression displayed a similar pattern to mPGES1; however, groups were not significantly different to one another (data not shown). Spinal expression of COX‐2 mRNA was not altered (compared with saline controls) in either the MIA vehicle or MIA MJN110 groups (Figure 4E).

Effects of repeated (1 mg·kg^−1^) MJN110 administration on MIA‐induced cartilage damage and inflammation were evaluated. Intra‐articular injection of MIA was associated with significant cartilage damage (MIA + vehicle group: 8.8 ± 2 vs. saline + vehicle: 0 ± 0) and inflammation (MIA + vehicle group: 1.7 ± 0.4 vs. saline + vehicle: 0 ± 0). Repeated administration of MJN110 did not significantly alter (worsen or improve) the extent of cartilage damage (9.50 ± 2.38) or inflammation (1.61 ± 0.49), compared with MIA + vehicle controls.

### Effects of MJN110 on brain levels of 2‐AG, AA and PGE2

**Figure 5:**
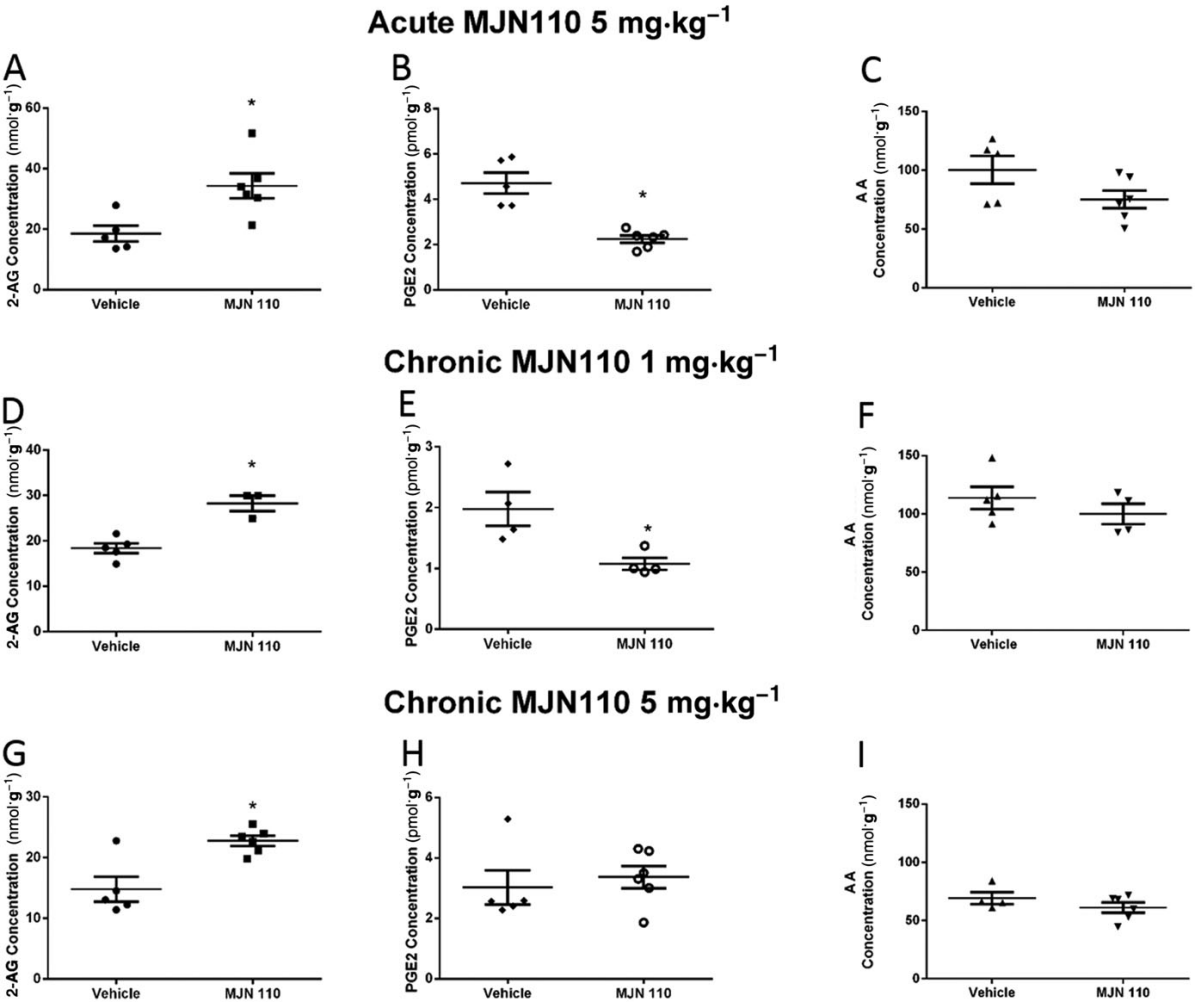
MJN110 elevates whole brain levels of 2‐AG and reduces levels of PGE2. Acute administration of MJN1110 (5 mg·kg^−1^) elevated levels of 2‐AG (A), reduced levels of PGE2 (B) but did not alter levels of AA levels (C). Chronic administration of MJN110 (1 mg·kg^−1^) elevated levels of 2‐AG (D), reduced levels of PGE2 (E) but did not alter levels of AA (F). Chronic administration of MJN110 (5 mg·kg^−1^) elevated levels of 2‐AG (G) but did not alter levels of PGE2 (H) or alter levels of AA (I). Data shown are means ± SEM; *n* = 4–6 rats per group. *P < 0.05, significantly different from vehicle; unpaired *t*‐tests.

MS analysis revealed a significant increase in brain levels of 2‐AG following acute administration of MJN110 (5 mg·kg^−1^) (Figure 5A) at 4 h post‐MJN110 administration. In the same samples, levels of PGE_2_ were significantly decreased (Figure 5B), and levels of AA were unaltered (Figure 5C). Repeated dosing of MJN110 (1 mg·kg^−1^) was associated with a significant increase in brain levels of 2‐AG (Figure 5D) and a significant reduction in levels of PGE_2_ (Figure 5E) but did not alter levels of AA (Figure 5F). Repeated dosing with MJN110 (5 mg·kg^−1^) was also associated with a significant increase in brain levels of 2‐AG (Figure 5G) but did not significantly alter levels of PGE_2_ (Figure 5H) or AA (Figure 5I).

## Discussion

In the present study, acute inhibition of MAG lipase with MJN110 significantly reversed MIA‐induced weight‐bearing asymmetry and MIA‐induced decrease in ipsilateral paw withdrawal thresholds. Effects were similar in magnitude to those of nimesulide in this model of OA pain (Sagar *et al.,* 2011). MJN110‐mediated reversal of MIA‐induced weight‐bearing asymmetry was dependent on CB_1_, and to a smaller extent CB_2_, receptor activation. In contrast, the ability of MJN110 to reverse MIA‐induced decreases in ipsilateral paw withdrawal threshold was blocked by the CB_2_, but not the CB_1_, receptor antagonist. This finding is consistent with the association of lowered hindpaw withdrawal thresholds with central sensitization and our earlier demonstration that the MIA model is associated with a significant increase in spinal CB_2_ receptor expression and alterations in CB_2_ receptor signalling as measured by spinal electrophysiology (Burston *et al.,* 2013). The magnitude of inhibition by the MAG lipase inhibitor was comparable to the anti‐nociceptive effects seen with FAAH inhibitors in acute inflammatory, neuropathic (Sasso *et al.,* 2012) and arthritic pain (Kinsey *et al.,* 2011).

Repeated treatment with the higher dose of MJN110 (5 mg·kg^−1^) resulted in a loss of anti‐nociceptive efficacy, whereas the lower dose (1 mg·kg^−1^) had sustained anti‐nociceptive effects on pain behaviour in the MIA model over the course of the study. It is, of course, possible that over longer periods of repeated dosing, tolerance may develop. Repeated treatment with MJN110 (1 mg·kg^−1^) did not alter the extent of MIA‐induced joint cartilage damage or joint inflammation, suggesting that blockade of MAG lipase does not alter this aspect of the model of OA, or possibly that by day 21 post‐MIA injection, knee pathology is already fully established and is no longer reversible.

Repeated treatment with MJN110 (1 mg·kg^−1^) significantly reduced expression of mPGES1 (the enzyme involved in the conversion of the COX metabolite, PGH_2_ to PGE_2_) in the dorsal horn of the spinal cord in MIA rats, compared with controls. These data plus the effects of MJN110 on hindpaw withdrawal thresholds, and the lack of effect on joint pathology, suggest that systemically administered MJN110 modulates OA pain behaviour via a spinal or supraspinal mechanism of action. Nevertheless, we have only evaluated the effect of two doses of MJN110 in this model, and therefore, a dose‐response relationship for analgesia or other parameters in this model has not been established.

To ascertain whether the effects of MJN110 were likely to be mediated by a change in bioactive lipids, we quantified brain levels of 2‐AG, PGE_2_ and AA. In keeping with a previous study (Parker *et al.,* 2015), acute administration of MJN110 (5 mg·kg^−1^) and chronic administration of the lower dose of MJN110 (1 mg·kg^−1^) were associated with increased brain levels of 2‐AG and a reduction in levels of PGE_2_ at 4 h post‐administration. Chronic administration of the higher dose of MJN110 (5 mg·kg^−1^) increased brain levels of 2‐AG but had no effect on PGE_2_ levels. None of the treatments altered brain levels of AA.

2‐AG‐induced analgesia has been described in models of acute and chronic pain (see Woodhams *et al.,* 2015). The differential effects of the two doses of MJN110 on OA pain behaviour reported here are consistent with the reported maximal and sub‐maximal blockade of MAG lipase by the two doses of MJN110, where only the former causes desensitization due to down‐regulation of CB_1_ receptors (Schlosburg *et al.,* 2010). Our demonstration of anti‐nociceptive tolerance of the higher dose of MJN110 supports the contribution of CB_1_ receptor‐mediated mechanisms to the anti‐nociceptive effects of MJN110 in this model of OA pain.

Recent studies have advanced understanding of the spinal mechanisms contributing to OA pain behaviour, demonstrating important contributions of dorsal horn neuronal excitability (Sagar *et al.,* 2010; Kelly *et al.,* 2013) and activation of spinal glia cells (Lee *et al.,* 2011; Sagar *et al.,* 2011; Ogbonna *et al.,* 2013; Yu *et al.,* 2013) (see references in Rani Sagar *et al.,* 2012). In the present study, repeated MJN110 treatment did not significantly inhibit MIA‐induced increases in GFAP mRNA in the dorsal horn of the spinal cord, which contrasts with the reported effects of an exogenous cannabinoid ligand selective for the CB_2_ receptor (Burston *et al.,* 2013). These differences between the effects of an exogenous CB_2_ ligand and those of MAG lipase inhibition on this spinal marker of astroglyosis may reflect differences in the study design (preventative treatment strategy vs. therapeutic herein) or differences between binding affinities of the CB_2_ ligand and that of 2‐AG (Huffman *et al.,* 1999; Gonsiorek *et al.,* 2000). Inhibition of MAG lipase reduced the AA substrate pool for PG production and decreased production of pro‐inflammatory mediators in the brain (Nomura *et al.,* 2011; Chen *et al.,* 2012; Lysenko *et al.,* 2014; Zhang *et al.,* 2015). In the present study, repeated treatment with the lower dose of MJN110 significantly reduced expression of spinal mPGES1 in MIA‐injected rats, compared with vehicle treatment and significantly decreased levels of PGE_2_ in the brain. Given that mPGES1 plays a major role in the production of pro‐inflammatory and pro‐nociceptive PGE_2_, our data suggest that the anti‐nociceptive effects of MJN110 may be, at least in part, mediated by the modulation of the PG system in the spinal cord and brain. Experimental constraints meant that spinal cord tissue was not available for MS analysis. With the assumption that the effects of MJN110 on MAG lipase in the spinal cord and brain are comparable, our demonstration that chronic MJN110 administration reduced levels of PGE_2_ in the brain further strengthens the evidence that the anti‐nociceptive effects of MJN110 are mediated by both an elevation of 2‐AG and activation of CB receptors and a reduction in synthesis (via decreased mPGES1 expression) or the production of PGE_2_ from metabolic substrates in the spinal cord.

In summary, our experiments have shown that MAG lipase inhibition had significant beneficial effects on established pain behaviour and was associated with a reduction in spinal pro‐inflammatory gene expression, an elevation of 2‐AG and a reduction in PGE_2_ in the brain. These new findings support the further investigation of MAG lipase as a target for the treatment of OA pain.

## Author contributions

J.J.B. performed the research, designed the research study, analysed the data and wrote the paper. P.I.M. performed the research, analysed the data and wrote the paper. S.S. analysed the data and wrote the paper. D.B. analysed the data and wrote the paper. M.J.N. contributed essential reagents or tools and wrote the paper. B.F.C. contributed essential reagents or tools and wrote the paper. D.A.W. wrote the paper. V.C. designed the research study and wrote the paper.

## Conflict of interest

The authors declare no conflicts of interest.

## Declaration of transparency and scientific rigour

This Declaration acknowledges that this paper adheres to the principles for transparent reporting and scientific rigour of preclinical research recommended by funding agencies, publishers and other organisations engaged with supporting research.
